# Understanding the impact of antimicrobial resistance on outcomes of bloodstream infections in low- and middle-income countries

**DOI:** 10.1371/journal.pmed.1004262

**Published:** 2023-07-12

**Authors:** Marlieke E. A. de Kraker

**Affiliations:** 1 Infection Control program, Geneva University Hospitals and Faculty of Medicine, Geneva, Switzerland; 2 WHO collaborating center, Geneva, Switzerland

## Abstract

Marlieke de Kraker discusses a systematic review and meta-analysis reporting the magnitude and consequences of bloodstream infections in low- and middle-income countries.

Antimicrobial resistance (AMR) is central to a large number of the 17 Sustainable Development Goals (SDGs) that all 191 UN Member States have agreed to achieve by 2030. Since 2020, AMR has been explicitly included as an indicator in SDG 3 on “Good health and well-being”.

As part of *PLoS Medicine*’s Special Issue dedicated to AMR, Kasim Allel and colleagues present the results of a systematic review and meta-analysis, providing a comprehensive overview of published articles estimating the burden of AMR among bacteremic patients hospitalized in low- and middle-income countries (LMICs) [1]. The authors screened nearly 12,000 records and assessed 1,000 full-text publications, to include 109 publications. They found that, in LMIC settings, AMR was associated with a 58% increase in bloodstream infection mortality rates, a doubling of the odds of ICU admissions, prolongation of hospital stay by a week, and an increase in direct medical costs of about 12,000 USD per case. They provide insights into the wide heterogeneity of reported burden estimates, by showing pooled results stratified for pathogen–drug resistance combinations, WHO region, and country income level. Their results underline the importance of having access to detailed, setting-specific data to accurately inform more granular AMR burden estimates.

It has been estimated that AMR could be responsible for 1.3 to 5 million deaths globally, the upper limit of which would place AMR in the top three causes of death after ischemic heart disease, stroke, and chronic obstructive pulmonary disease [2]. In this same study, it was determined that the highest burden of AMR was found in low-income settings; highest death rates were estimated for sub-Saharan Africa and South Asia. However, this is based on modeling [3], and the authors indicated that the sparsity of data from many LMICs was an important limitation of their work. The current study by Allel et al. [1] appears to contradict the statement from Murray et al. [2] regarding the scarcity of data on AMR attributable mortality from LMICs. However, diving into the details of the systematic review, the authors show that a substantial number of the available studies were carried out in upper-middle income countries (96/109), of which the majority were conducted in one country (49/96 studies from China). Only 1 study was conducted in a low-income country, and only 4 studies included were from the African continent. Moreover, the reported, pooled estimates could only be based on crude data; case-fatality rates were directly compared between patients with drug-resistant infections and patients with drug-susceptible infections, even though patients with drug-resistant infections frequently have underlying comorbidities that increase their risk of death independent of the presence of a drug-resistant infection [4,5]. Excess length of stay could only be assessed by comparing overall length of stay between patients with drug-resistant and drug-susceptible infections, ignoring the commonly longer admission period prior to infection onset for patients with drug-resistant infections [6]. These biases have, most likely, resulted in an overestimation of the impact of AMR.

There is little high-quality, empirical data to support the notion that the largest burden of AMR can be found in LMICs. In a recent, prospective, observational study (MBIRA), including 8 hospitals from as many sub-Saharan African countries, Enterobacterales bloodstream infections greatly increased mortality, but the drug resistance profile of the Enterobacterales was not associated with an increased mortality risk [5]. This may partly be explained by healthcare seeking behavior in LMIC settings, where, in certain settings, only the most severe cases of drug-susceptible bloodstream infection are seen at the hospital. However, a more likely reason is the limitations of healthcare services due to inadequate resources. In these settings, the added impact of drug-resistance for severe infections may be minimal, especially for highly specialized care, like neonatal care. Access to antibiotics is another factor that could influence the impact of drug resistance: in the hospitals participating in MBIRA, there was a marked variation in access to antibiotics over time [7]. Due to lack of access to antibiotics, patients either receive no antibiotic or inappropriate antibiotics, or resort to buying antibiotics from external, private pharmacies. Consequently, there is a delay in administration of appropriate treatment, and patients may receive counterfeit drugs, with no or limited anti-bacterial activity. This underlines the importance of general infection prevention and control measures, improved antibiotic access, and higher quality care for bloodstream infections, irrespective of drug resistance [5,8,9].

At the moment, there are a number of studies ongoing in LMIC settings, like BARNARDS II [10], focusing on neonatal sepsis; BALANCE [10], comparing the impact of drug-resistant bloodstream infections across income settings; and ACORN [11], applying the WHO AMR attributable mortality protocol [12] to determine the impact of drug-resistant bloodstream infections. These projects will, hopefully, provide more insights into the true burden of AMR in LMICs and offer guidance with regards to the most important pathogens, patient risk groups, and settings for which prevention and intervention strategies should be considered.

There is a pressing need for more high-quality, detailed data to better understand the burden of AMR in LMICs and beyond. However, this should not impede any investments to improve infection prevention and control, diagnostic and antimicrobial stewardship and supportive care for patients with serious bacterial infections in LMIC settings. These investments are urgently needed to improve patient outcomes and to make the SDGs attainable by 2030. The UN General Assembly High-Level meeting on AMR in September 2024 could play an important role by setting relevant targets and fostering collective action to reduce the global burden of drug-resistant and drug-susceptible infections.
